# Teasaponin Ameliorates Murine Colitis by Regulating Gut Microbiota and Suppressing the Immune System Response

**DOI:** 10.3389/fmed.2020.584369

**Published:** 2020-12-10

**Authors:** Huan Yang, Rui Cai, Ziyan Kong, Ying Chen, Chen Cheng, Suhua Qi, Bing Gu

**Affiliations:** ^1^Xuzhou Key Laboratory of Laboratory Diagnostics, Medical Technology School of Xuzhou Medical University, Xuzhou, China; ^2^Department of Laboratory Medicine, Affiliated Hospital of Xuzhou Medical University, Xuzhou, China

**Keywords:** inflammatory bowel disease, immune response, gut microbiota, intestinal permeability, teasaponin

## Abstract

**Background:** Dietary intervention is an exciting topic in current research of inflammatory bowel disease (IBD). The effect of teasaponin (TS) on IBD has not been fully elucidated. Here, we aim to investigate the intestinal anti-inflammatory activity of TS in a dextran sodium sulfate (DSS)-induced colitis mouse model and identify potential mechanisms.

**Methods:** We applied TS to mice with DSS-induced colitis and then monitored the body weight, disease activity index (DAI) daily. When sacrificed, the intestinal permeability was measured. The analysis of mucin and tight junction proteins was conducted. We detected the inflammatory cytokines, the immune cells and related inflammatory signaling pathways. In addition, the gut microbiota were analyzed by 16S rRNA sequencing and we also performed fecal microbiota transplantation (FMT).

**Results:** It showed that TS ameliorated the colonic damage by lowering the DAI, prolonging the colon length, reducing inflammatory cytokines and improving the mucus barrier. Parallel to down-regulation of the inflammatory cytokines, the fecal lipocalin 2, p-P65, p-STAT3, and neutrophil accumulation were also decreased in TS-treated mice. Microbiota characterization showed that *Campylobacteria, Proteobacteria, Helicobacter*, and *Enterobacteriaceae* were the key bacteria associated with IBD. In addition, TS could reverse the *Firmicutes*/*Bacteroidetes* (F/B) ratio and increase the beneficial bacteria, including Akkermansia and Bacteroides. TS ameliorated DSS-induced colitis by regulating the gut microbiota, and the gut microbiota could regulate gut inflammation.

**Conclusions:** These studies demonstrated that TS ameliorated murine colitis through the modulation of immune response, mucus barrier and gut microbiota, thus improving gut dysbiosis. In addition, the gut microbiota may play an important role in regulating the host's innate immune system, and the two coexist and are mutually beneficial. We provide a promising perspective on the clinical treatment of IBD.

**Graphical Abstract:**
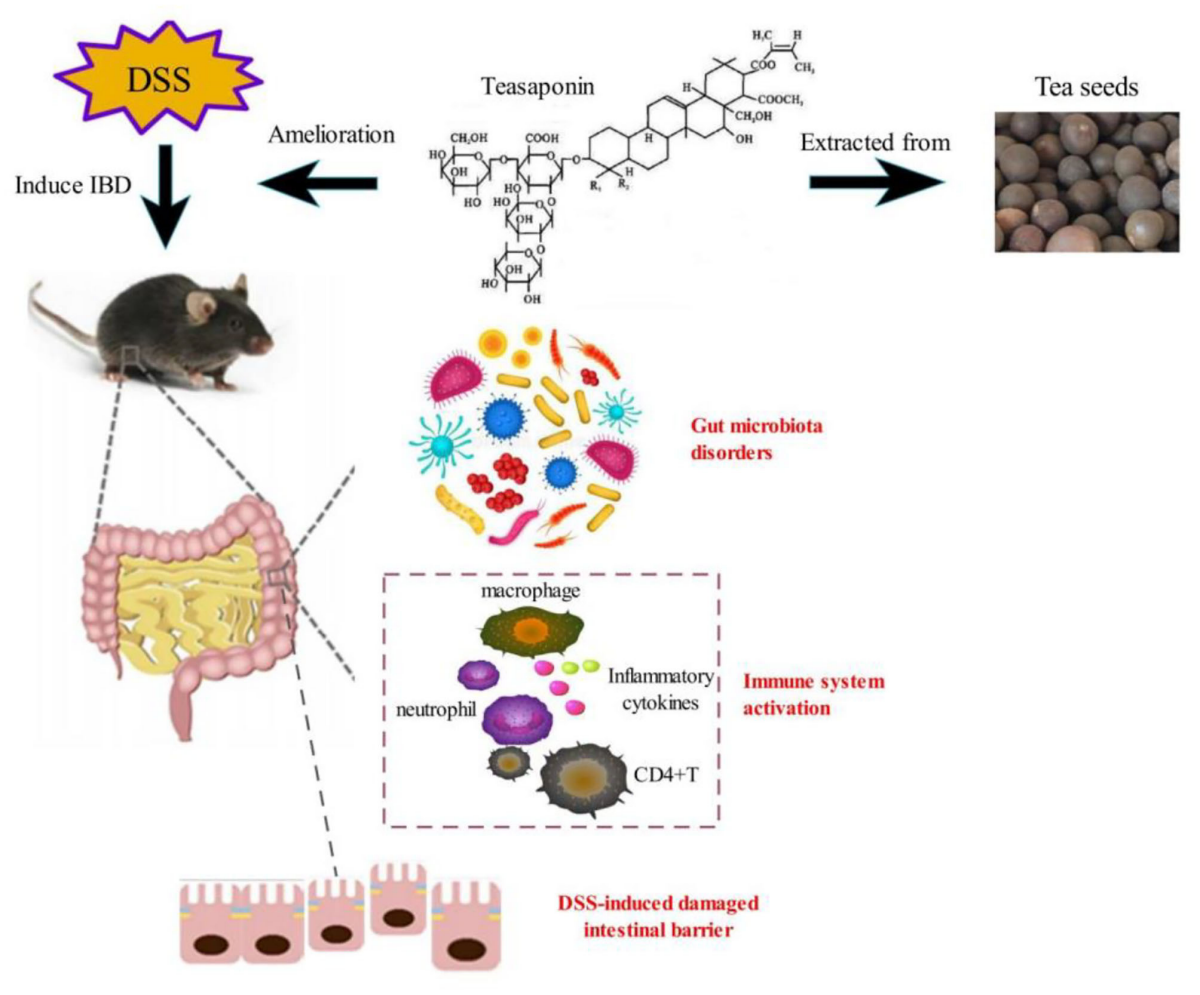


## Introduction

Ulcerative colitis (UC) is one form of IBD with a substantial impact on the quality of life of affected persons. The incidence of UC is not only high in Western developed countries but on the rise in China and other Asian countries in recent years (1, 2). The main symptoms include diarrhea, bellyache, tenesmus and even fever and weight loss. Inflammation is superficial and continuous, confined to the mucosa and submucosa. Notably, UC patients are more likely to develop colorectal cancer (3). There is no determinate conclusion about the pathogenesis of UC, knowing that it may be caused by a combination of host genetics, immune disorders, intestinal microbes, intestinal barrier dysfunction and so on. Numerous clinical data and animal experiments have demonstrated that UC is often accompanied with intestinal immunity dysfunction and imbalance of gut microbiota (4–7).

Common drugs currently available for IBD include aminosalicylates, corticosteroids, thiopurines and biological agents, but they all have serious adverse effects, which urges researchers to develop more effective and less toxic alternative drugs for the treatment of IBD (8). Many studies have focused more attention on natural and functional materials such as polyphenols and terpenoids (7). Terpenoids are natural chemical ingredients with a wide variety of biological activities, especially anti-inflammatory and anticancer potential (9).

Tea has been cultivated for thousands of years and used for beverage and medicinal purposes. Recently, research on tea has shown that tea possesses anti-inflammatory properties, and can improve microbiota and cognition (10). TS is major active component of tea, chemically belonging to oleanane-type pentacyclic triterpene saponins (11). TS has anti-inflammatory, analgesic, anti-osmotic and other pharmacological activities. Different mechanisms have been proposed to participate in the beneficial effects of TS, such as reducing inflammatory cytokines by inhibiting the main inflammatory regulators including NF-κB, which is known to play a crucial role in the pathogenesis of IBD (11). Wang et al. also demonstrated that TS could reduce high-fat (HF)-induced endotoxemia and macrophage accumulation in the colon. In addition, TS could improve the gut microbiota alteration caused by HF diet and limit the growth of unfavorable gut microbiota (10).

However, little information about the effect of TS on IBD is available. We aimed to investigate the intestinal anti-inflammatory activities and the effect on the gut microbiota of TS in a DSS-induced colitis mouse model. It was found that the effect was associated with decreased immune response and inflammatory cytokines. In addition, the effect on the gut microbiota demonstrated that interventions targeting the microbiota are a potential path for IBD treatment. Our results suggested that TS-associated microbiota was consistent with immune response, and proved that TS may be a potential therapeutic target for patients with IBD.

## Methods

### Establishment of the DSS-Induced Colitis Mouse Model

All animal experiments were performed according to the standards in the Guide for the Care and Use of Laboratory Animals (Institute of Laboratory Animal Resources of National Research Council, United States). All mouse studies were evaluated by the Laboratory Animal Ethics Committee of Xuzhou Medical University (IACUC number: 201908A005), Xuzhou, China. We made every effort to minimize animal suffering and to reduce animals used.

Male C57BL/6J mice aged 6–8 weeks were purchased from Vital River Laboratory Animal Technology Co., Ltd. (Zhejiang, China). All mice were housed under specific pathogen-free conditions at the Experimental Animal Center of Xuzhou Medical University (Xuzhou, China).

The mice were randomly divided into three different groups: NC group, DSS group, and TS treatment (DSS+TS) group. Animal in NC and DSS group received sterile water (100 μL/day) orally, and those in DSS+TS group received TS (400 mg/kg/day) for 16 days by oral gavage. At day 8, colitis was induced by 2% DSS (36–50 kDa, MP Biomedicals, Canada) in the drinking water for 8 days. Teasaponin is extracted from Camellia oleifera seeds. Teasaponin belongs to the triterpene saponins. It has strong foaming, emulsifying, dispersing and wetting effects. Teasaponin (60%≥, C_57_H_90_O_26_, molecular weight 1,200) was purchased from the Aladdin Chemistry Co., Ltd. (Shanghai, China).

The score of DAI was calculated by body weight, the presence of blood in the feces and stool consistency. The following scores were given: (i) weight loss (0: no loss, 1: 1–5%, 2: 5–10%, 3: 10–20%, 4:>20%), (ii) bleeding (0: no blood, 1: hemoccult +, 2: hemoccult + and visual pellet bleeding, 4: gross bleeding with blood seen around the anus); and (iii) stool consistency (0: normal, 2: loose stool, 4: diarrhea). On day 16, the mice were sacrificed and the colon tissue and cecal contents were collected. The middle of the colon was fixed in 4% formaldehyde for histological observation. The remaining colon and cecal contents were stored at −80°C.

### Enzyme-Linked Immunosorbent Assays (ELISA)

The upper colon tissue was weighed, homogenated with phosphate buffer solution and centrifuged at 10,000 rpm/min for 10 min. The extracted total protein in the supernatant was collected to measure the expression of TNF-α, IL-1β, IL-6, IL-17, and IL-33 by ELISA kits (Neobioscience, Tianjin, China), according to the manufacturer's instructions. The results were read with a microplate reader (Bio-Rad iMARK, USA) at 450 nm.

### RNA Extraction and RT-PCR

The total RNA from the lower colon was extracted using the Total RNA Extraction Kit (Solarbio, Beijing, China) according to the manufacturer's protocol and purified with lithium chloride described by Viennois et al. (12). mRNA was quantified with UltraSYBR Mixture (Cwbio, Beijing, China) and specific primers by qPCR (Roche Light Cycler 480 II, USA) according to the protocol of the manufacturer. Gene expression was normalized to β-actin and calculated by 2^−ΔΔCt^. The primers were: β-actin: 5′ GGCTGTATTCCCCTCCATCG3′ and 5′CCAGTTGGTAACAATGCCATGT3′; TNF-α: 5′GA TCGGTCCCCAAAGGGATG3′ and 5′TTTGCTACGACGTGGGCTAC3′; IL-1β: 5′TGCCACCTTTTGACAGTGATG3′ and 5′ATGTGCTGCTGCGAGATTTG3′; IL-6: 5′AGACAAAGCCAGAGTCCTTCAG3′ and 5′GAGCATTGGAAATTGGGGTAGG3′; IL-17: 5′ TTTAACTCCCTTGGCGCAAAA3′ and 5′CTTTCCCTCCGCATTGACAC3′; IL-33: 5′ TCCAACTCCAAGATTTCCCCG3′ and 5′CATGCAGTAGACATGGCAGAA3′.

### Histological and Morphometric Analysis

The middle of the colon was fixed in 4% formaldehyde. Histological sections (4 μm) were stained with HE for morphometric examination. The score was according to the degree of inflammatory infiltration (0–5), crypt injury (0–4), ulcer (0–3), and the absence of edema (0 or 1), as described by Stillie et al. (13).

The slides were deparaffinized and then incubated with F4/80 antibody (Abcam, 1:100), Ly6G antibody (Abcam, 1:100), CD4 antibody (Servicebi, 1:100), Muc2 antibody (Proteintech, 1:100), Claudin-1 antibody (Proteintech, 1:100) and ZO-1 antibody (Proteintech, 1:100) in blocking buffer overnight at 4°C overnight, followed by incubation with Alexa Fluor 594-labeled second antibody (Proteintech) for 1 h. The sections were then stained with DAPI for nuclear counterstaining. The images were observed by fluorescence microscopy (Olympus IX71, Tokyo, Japan).

The slides were deparaffinized, stained with Phospho-NF-κB p65 (Servicebio, 1:100) and Phospho-STAT3 antibody (Abcam, 1:100) overnight at 4°C, incubated with a rabbit HRP polyclonal antibody for 1 h at room temperature, and finally visualized with DAB (VECTOR).

### Microbial Community Analysis by 16S rRNA Gene Sequencing

Mice cecal contents were collected and stored at −80°C after snapping frozen in liquid nitrogen. The DNA of total bacteria in mice cecal contents was extracted with QIAamp^®^ Fast DNA Stool Mini Kit. For 16S rRNA gene sequencing, the DNA samples were sent to Microbiology Division, Meiji Biomedical Technology Co., Ltd. (Shanghai, China) under −20°C preservation and dry ice conditions. High fidelity PCR was utilized to amplify bacterial 16S rRNA hypervariable region 3 (V3) and hypervariable region 4 (V4) with the primers. High-throughput sequencing was performed on Illumina Miseq platform with 2 × 250 bp paired-end method after quantifying, mixing and quality checking the library. All the results were based on sequenced reads and operational taxonomic units (OTUs).

### FITC-dextran Assay for Detecting Intestinal Permeability

On the 16th day of the TS treatment, the mice were deprived of water for 2 h, and then each mouse was given a 40 mg/100 g FITC-dextran solution. After intragastric administration for 4 h, blood was taken from the mouse eyeballs, centrifuged at 4°C, 3,000 rpm/min for 15 min, and serum was obtained. The fluorescence intensity of FITC was detected at 485/530 nm with a fluorescence microplate reader, and the concentration of FITC-dextran in the sample was calculated according to the standard curve.

### Fecal Microbiota Transplantation

First, mice are randomly divided into donor group and recipient group. For donor group, animal in NC and DSS group received sterile water (100 μL/day) orally, and those in DSS+TS group received TS (400 mg/kg/day) for 16 days by oral gavage. At day 8, colitis was induced by 2% DSS in the drinking water for 8 days. At day 10, in order to establish a sterile mouse model, recipient mice were given a mixture of antibiotics (ampicillin 1 mg/mL, neomycin 1 mg/mL, metronidazole 1 mg/mL and vancomycin 0.5 mg/mL) in drinking water and oral gavage (200 μL/day) for 5 days. At day 15, stopped antibiotics and prepared fecal microbiota transplantation. At day 16, 200 mg per mouse of feces from donor mice was dissolved in 1 ml of 0.05% cysteine solution, stirring well and filtering with 100 μl screen to obtain fresh fecal filtrate, which was administered to recipient mice by orally gavage (200 μL/mouse) for 5 consecutive days. Body weight and DAI were observed every day, and feces were collected to test the colonization efficiency of fecal microbiota.

### The Detection of Fecal Lipocalin 2

The feces were weighed, homogenated with phosphate buffer solution and centrifuged at 10,000 rpm/min for 10 min. The extracted total protein in the supernatant was collected to measure the expression of lipocalin 2 (LCN2) by ELISA kits (Abcam), according to the manufacturer's instructions. The results were read with a microplate reader (Bio-Rad iMARK, USA) at 450 nm.

### The Myeloperoxidase Assay in Tissues

The upper colon tissue was weighed, homogenated with phosphate buffer solution and centrifuged at 10,000 rpm/min for 10 min. The extracted total protein in the supernatant was collected to measure the expression of Myeloperoxidase (MPO) by ELISA kits (Abcam), according to the manufacturer's instructions. The results were read with a microplate reader (Bio-Rad iMARK, USA) at 450 nm.

### Flow Cytometry Analysis of Colon Tissues

The colon tissues were sliced with a scalpel into many small pieces and digested for 30 min at 37°C with 0.5 mg/ml collagenase (Sigma-Aldrich) and 100 g/ml DNAse I in PBS. After digestion, tissues were mashed and single cell suspensions were filtered through a 70 μm nylon mesh and washed with PBS. Then we separated mononuclear cells with 40% percoll. Single cell suspensions were incubated with following antibodies: anti-CD11b conjugated to APC (eBioscience, San Diego, CA, USA), anti-Ly6G conjugated to PE (eBioscience), anti-F4/80 conjugated to FITC (eBioscience, San Diego, CA, USA), anti-CD45 conjugated to APC (eBioscience, San Diego, CA, USA), anti-CD4 conjugated to PE (eBioscience), anti-CD3 conjugated to FITC (eBioscience), Cells were analyzed on a FACSCanto II Flow Cytometer (BD Biosciences) using the Flow Jo software (FlowJo, Ashland, OR, USA).

### Statistical Analysis

Statistical analysis was performed using GraphPad Prism (GraphPad Software). Data are expressed as the mean ± SEM. Comparisons between two groups were assessed using a student *t*-test or Mann-Whitney test depending on whether the data were normally distributed. Statistical significance between multiple groups was tested using a one-way multiple analysis of variance (ANOVA) or Kruskal-Wallis. The level of statistical significance was set at *p* < 0.05.

## Results

### TS Suppressed DSS-Induced Colitis in Mice

**Figure 1:**
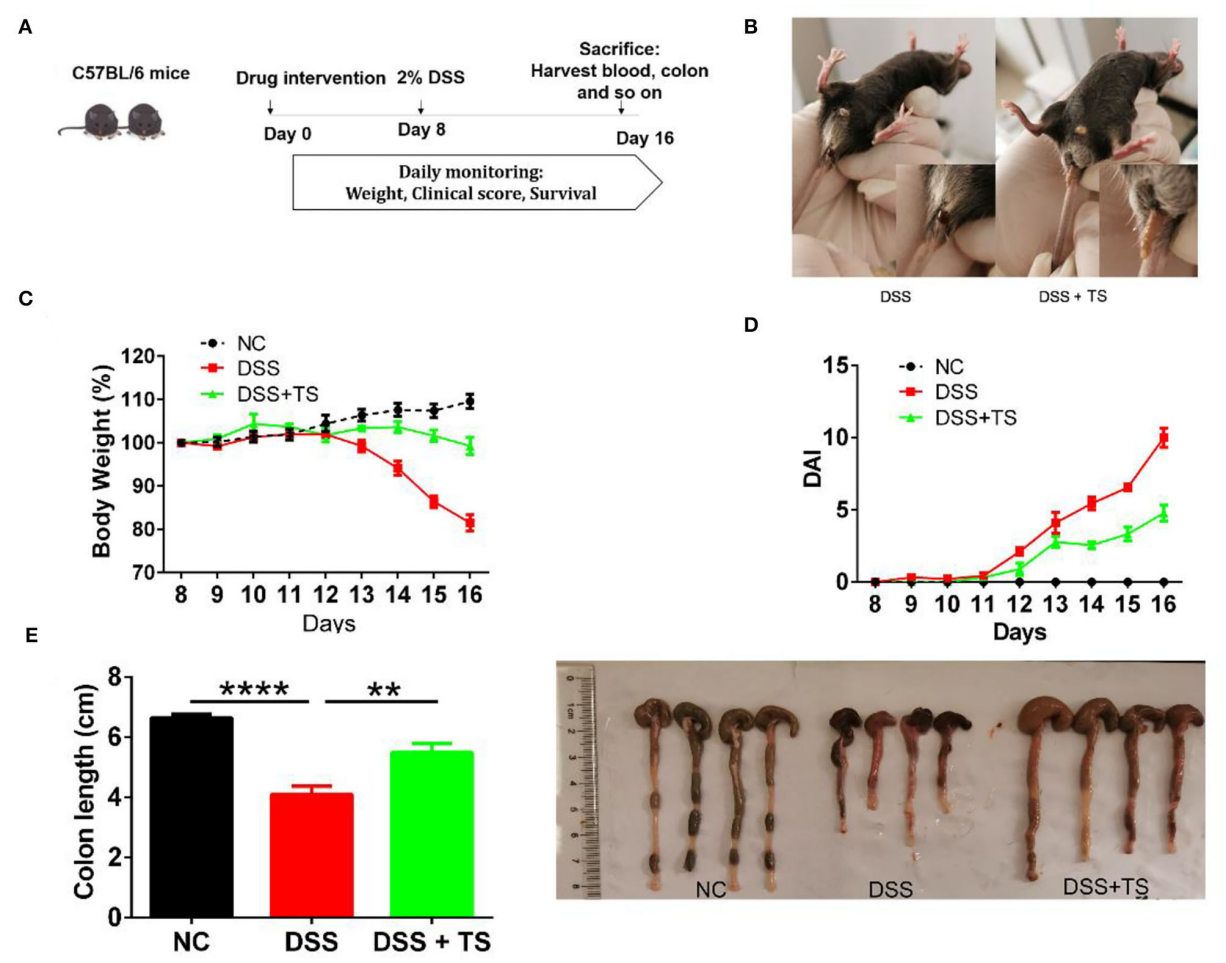
TS ameliorates DSS-induced colitis in mice. **(A)** Flow diagram; **(B)** symptoms and fecal characters; **(C)** body weight; **(D)** DAI score; **(E)** colon length. Data are expressed as the mean ± SEM (*n* = 9). Statistical significance was determined with one-way ANOVA followed by Tukey test, and *p*-values are as follows: ^**^p < 0.01, ^****^p < 0.0001.

To clarify the role of TS in DSS-induced colitis, we treated the mice with TS for 16 days and induced murine DSS colitis by administrating 2% DSS in drinking water from day 8 (Figure 1A). The administration of 2% DSS for 8 days produced an increase in DAI scores, due to decreased body weight and diarrheic/bleeding feces. However, the mice treated with TS showed a reduced DSS damage, including body weight loss and a lower incidence of diarrheic/bleeding feces (Figures 1B,C), resulting in lower DAI scores in the DSS+TS group compared with the DSS group (Figure 1D). In addition, the macroscopic observation of the colonic segments demonstrated the ameliorative effect of the DSS+TS group. TS prevents colon shortening compared with that of the DSS group (Figure 1E).

### TS Improved the Colonic Mucus Barrier

**Figure 2:**
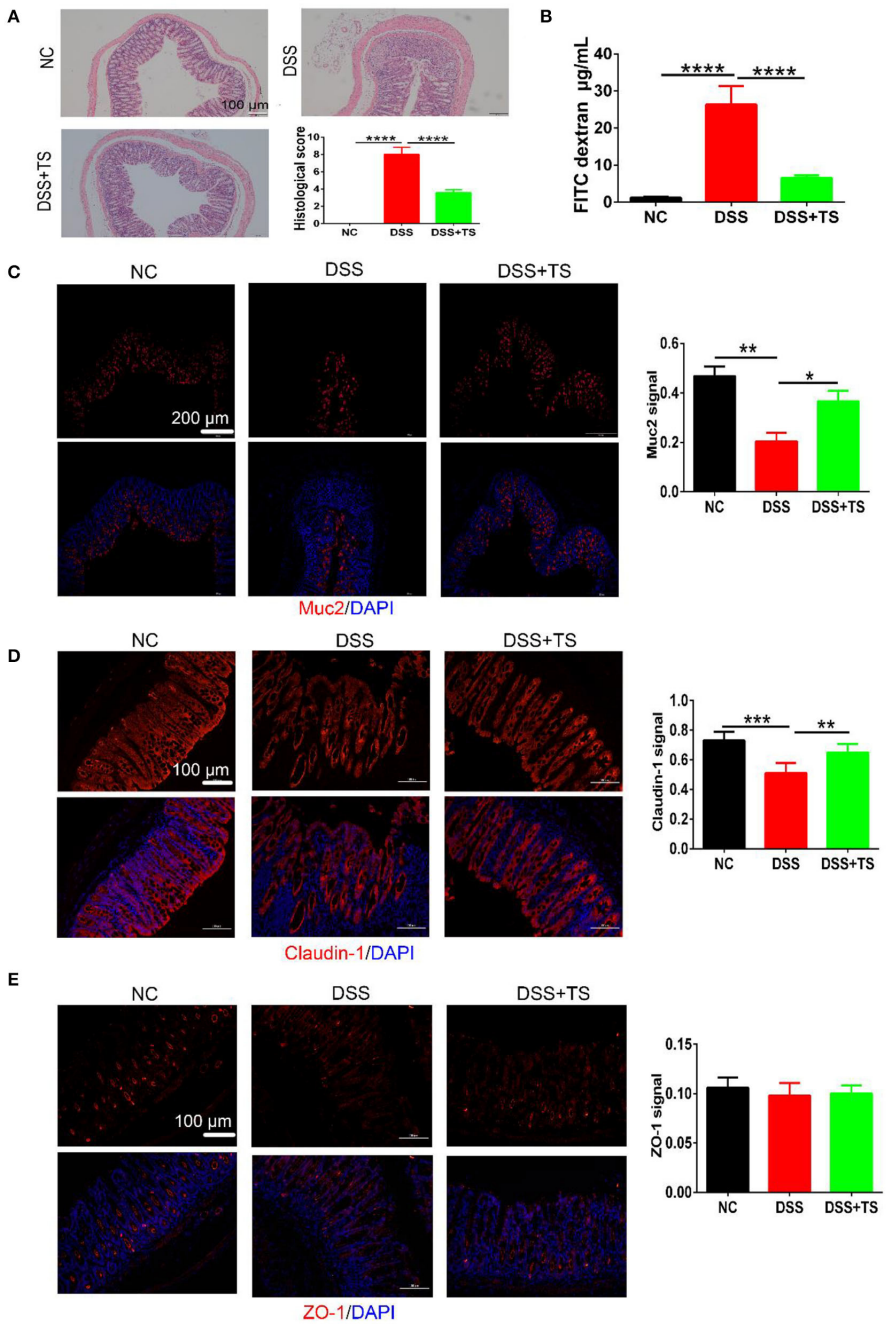
The effect of TS on the mucus barrier damaged by DSS. **(A)** Representative images of H&E stained histological sections and histopathology scores; **(B)** detection of FITC-dextran in serum; **(C–E)** representative images of immunofluorescence of MUC2, Claudin-1, and ZO-1. The mucins and tight junction proteins are stained red, and nuclei are counterstained blue. Quantitative analysis of Muc2, Claudin-1, and ZO-1 signal was using Image-Pro Plus. Data are expressed as mean ± SEM (*n* = 7), statistical significance was determined with one-way ANOVA followed by Tukey test, and *p*-values are as follows: ^*^p < 0.05, ^**^p < 0.01, ^***^p < 0.001, ^****^p < 0.0001.

To evaluate the level of the colonic damage under the microscope, the histopathological scores of the representative H&E stained histological sections were measured (Figure 2A). The colon of the DSS group showed moderate-severe edema and inflammation in the mucosal and muscular layer. Besides, there were more than two-thirds of disappeared crypts and incomplete epithelial cells in the DSS group. The DSS+TS group showed mild edema and inflammation, and the crypts disappeared by about one third. The histological score manifested a significant remission in the DSS+TS group compared with the DSS group.

To further characterize the damage to the epithelial layer, intestinal permeability was quantified by oral administration of FITC-dextran to mice on day 8 of DSS treatment, and fluorescence intensity in the serum was measured (Figure 2B). The diffusion of FITC-dextran from the intestine to the serum after TS treatment was significantly decreased compared with the DSS group, suggesting that TS improved the ability of maintaining the function of the colonic mucus barrier during DSS administration.

Knowing that another crucial component of maintaining the function of the intestinal mucus barrier is the formation and distribution of some proteins between epithelial cells including mucins and tight junction proteins, we observed the expression and distribution of these proteins in the colon, and the results of immunofluorescence analysis of MUC2, ZO-1, and Caludin-1 were shown (Figures 2C–E). In the NC group, crypts were filled with MUC2 except the basilar part of the mucosal layer. The distribution of MUC2 was narrowed along with the disappearance of crypts in the DSS group, manifesting a severe colonic damage induced by DSS. While, the distribution of MUC2 in the DSS+TS group was across the mucosal layer, suggesting the compensatory reaction to the slight inflammation. Claudin-1 and ZO-1, both known as the tight junction proteins, were arrayed compactly and orderly in the NC group. The distribution of Claudin-1 and ZO-1 was dispersed and hollow in the DSS group. Conversely, TS improved the damage induced by DSS administration.

### TS Attenuated Intestinal Inflammation

**Figure 3:**
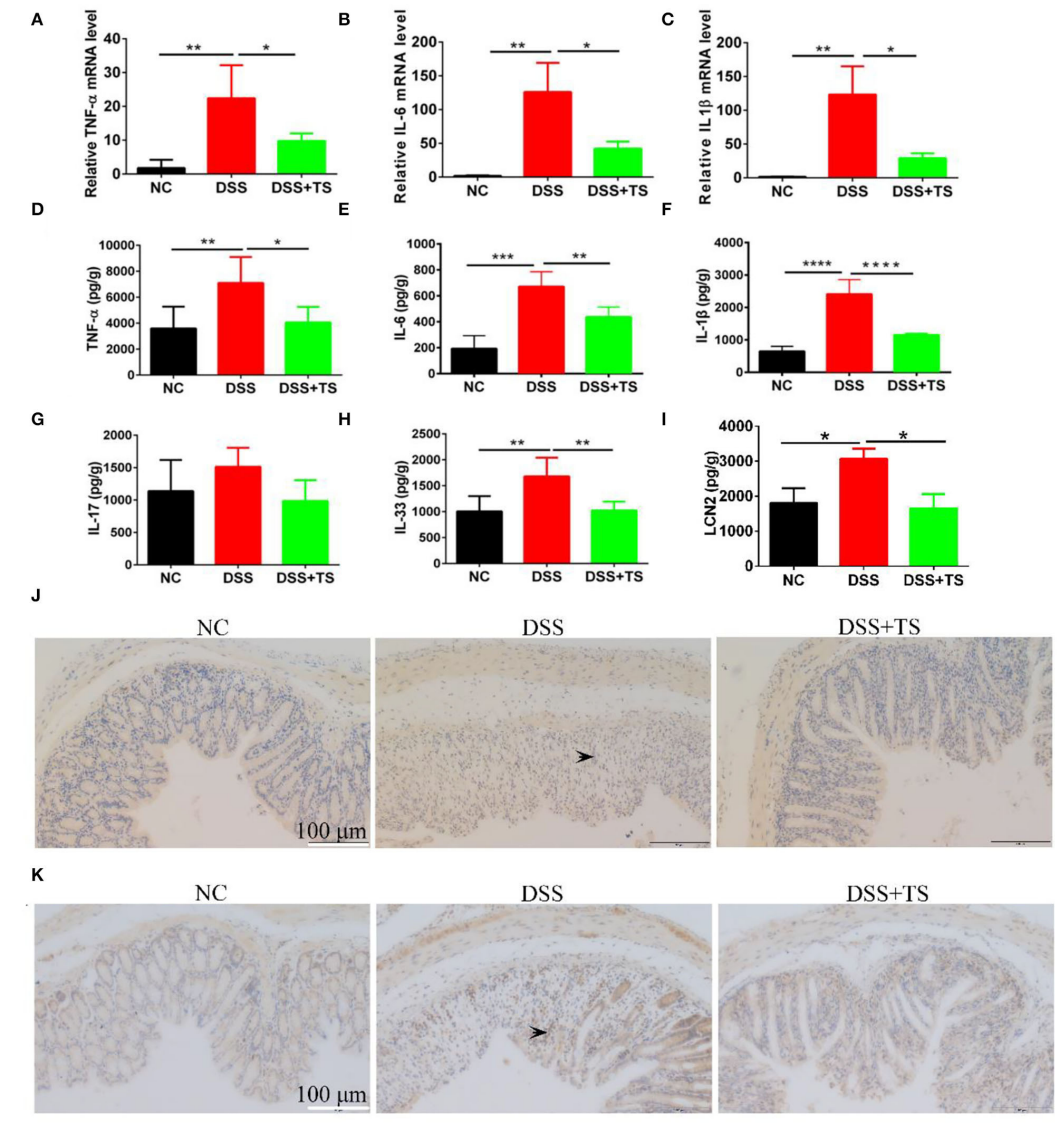
The effect of TS on the expression of inflammatory signal caused by DSS. **(A–C)** The mRNA levels of TNF-α, IL-6, and IL-1β by RT-qPCR; **(D–F)** the protein levels of TNF-α, IL-6, and IL-1β by ELISA; **(G,H)** the protein levels of IL-17 and IL-33 by ELISA; **(I)** the levels of fecal LCN2 by ELISA; **(J)** representative immunohistochemistry images of p-STAT3 in the colon tissues. **(K)** representative immunohistochemistry images of p-P65 in the colon tissues. The arrows indicate the positive staining p-STAT3 and p-P65. Data are expressed as mean ± SEM (*n* = 5), statistical significance was determined with one-way ANOVA followed by Tukey test, and *p*-values are as follows: ^*^p < 0.05, ^**^p < 0.01, ^***^p < 0.001, ^****^p < 0.0001.

To investigate whether TS resulted in the changes in inflammatory response, levels of TNF-α, IL-1β, and IL-6 were quantified in the colon tissue using RT-qPCR and ELISA. Compared with the NC group, the mRNA levels of these pro-inflammatory cytokines were increased markedly in the DSS group. While the mRNA levels of these observed cytokines were significantly reduced in DSS+TS group (Figures 3A–C). Meanwhile, the protein levels of these inflammatory cytokines manifested the same results (Figures 3D–I). As a frequently used marker to measure DSS induced intestinal inflammation, we inclued the fecal lipocalin 2 ELISA data. The fecal lipocalin 2 showed a significant decrease in the DSS+TS group compared with the DSS group in Figure 3K. Generally, TS reduced the levels of pro-inflammatory cytokines in the colon treated with DSS. Immunohistochemistry (IHC) staining analysis demonstrated that phospho-STAT3 existed in the DSS group, especially localized the place with severe inflammation. In contrast, phospho-STAT3 was not detected in the DSS+TS group (Figure 3J). Here we focused on phospho-P65 (NF-kB signaling), which is associated with the development and progression of inflammation. IHC staining showed that TS inhibited P65 phosphorylation level relative to the DSS group (Figure 3K).

### TS Ameliorated Immune System Disorder Induced by DSS

**Figure 4:**
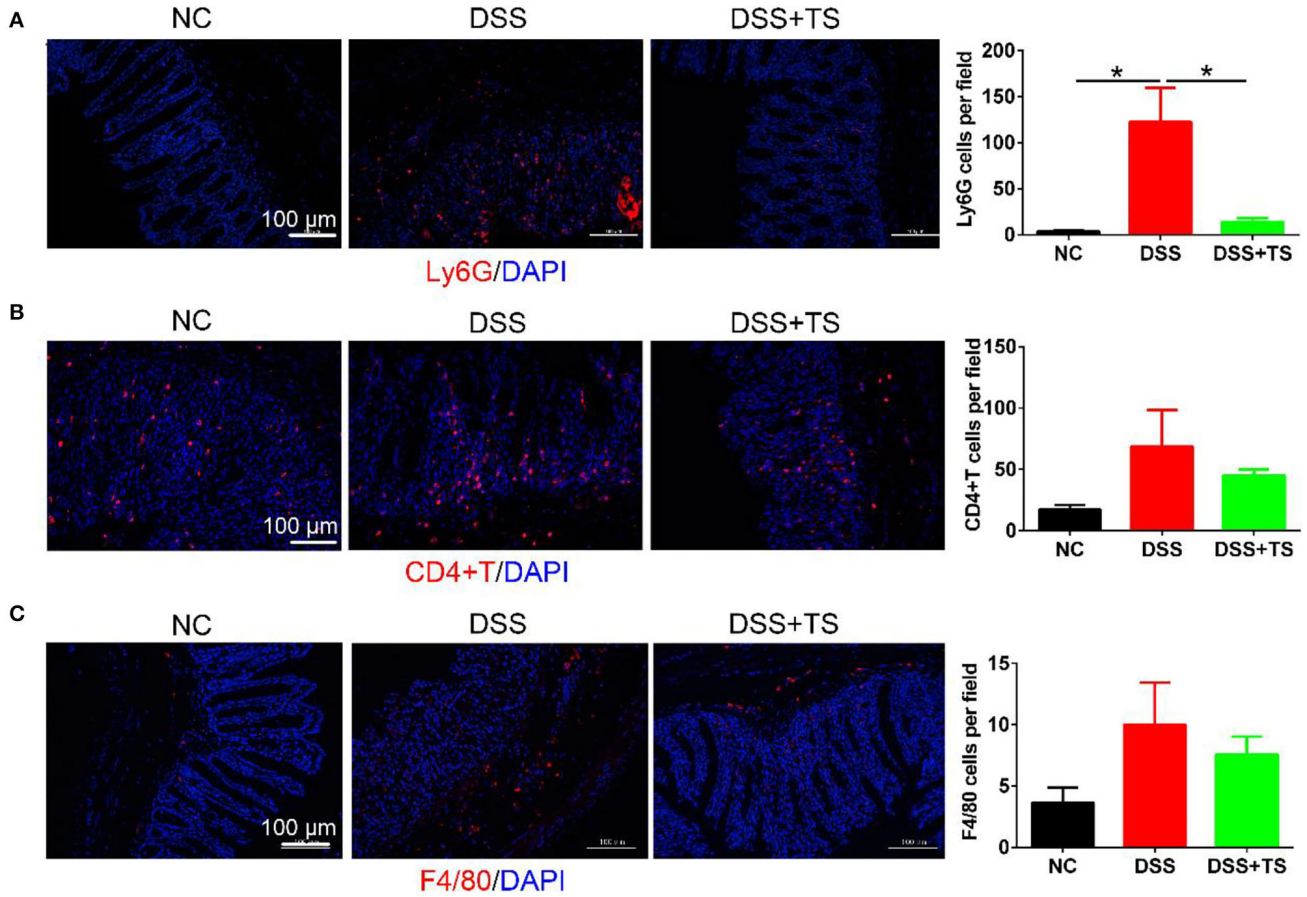
Effects of TS on the immune system disorder analyzed by immunofluorescence. **(A)** Representative immunofluorescence staining images of neutrophils (Ly6G) and mean positive cells counted per field in the colon tissues (*n* = 5); **(B)** representative immunofluorescence staining images of T cells (CD4+T) and mean positive cells counted per field in the colon tissues (*n* = 5); **(C)** representative immunofluorescence staining images of macrophage (F4/80) and mean positive cells counted per field in the colon tissues (*n* = 5); The immune cells (Ly6G, CD4+T, F4/80) are stained red, and nuclei are counterstained blue. Data are expressed as mean ± SEM, statistical significance was determined with one-way ANOVA followed by Tukey test, and *p*-values are as follows: ^*^p < 0.05.

**Figure 5:**
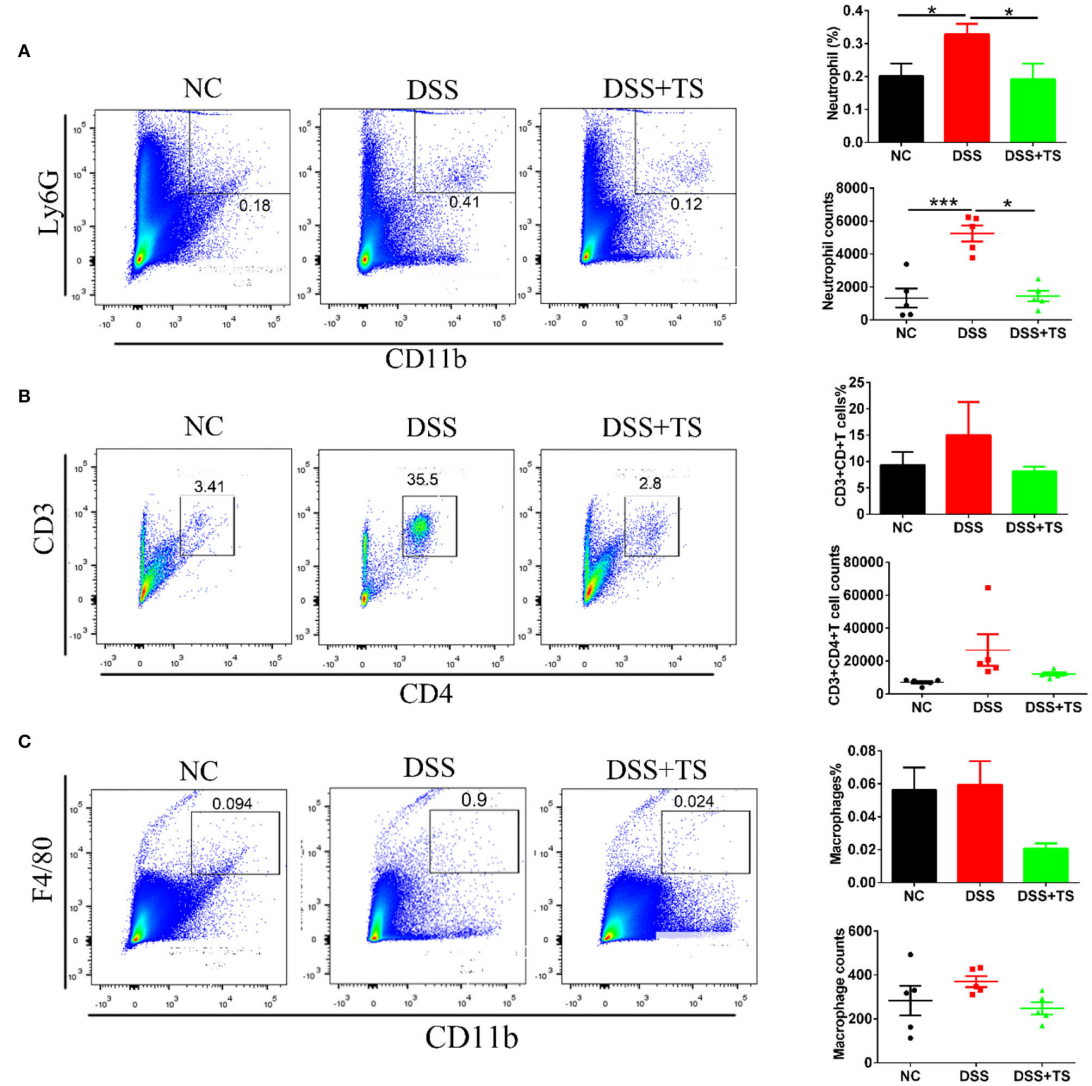
Effects of TS on the immune system disorder analyzed by flow cytometry. Percentages and counts of neutrophils **(A)**, CD4+T cells **(B)** and macrophages **(C)** in lamina propria of colon (*n* = 5). Data are expressed as mean ± SEM, statistical significance was determined with one-way ANOVA followed by Tukey test, and *p*-values are as follows: ^*^p < 0.05, ^***^p < 0.001.

Elevated levels of inflammatory cytokines are markers of immune-related disorder. In general, the release of inflammatory factors recruits immune cells. Therefore, we investigated relevant immune cells in the colon tissue by immunofluorescence (Figures 4A–C) and flow cytometry analysis (Figures 5A–C). Ly6G, a marker for neutrophils, was increased in the DSS group, but the TS reduced the recruitment. We used the CD45 marker to separate white blood cells first, and CD3CD4 double positive represents CD4+T cells. CD3+CD4+T cells were also increased in the DSS group compared with the other groups. The expression was decreased in the DSS+TS group, even though the difference was not statistically significant. F4/80 is a marker for macrophages, which gives rise to pro-inflammatory cytokines. It was elevated in the DSS group compared with the other groups. The expression was reduced in the DSS+TS group, but the difference was not statistically significant. Therefore, TS mainly played a role in recruiting fewer neutrophils to reduce inflammation.

### Impacts of TS on the Gut Microbiota in DSS-Induced Colitis

**Figure 6:**
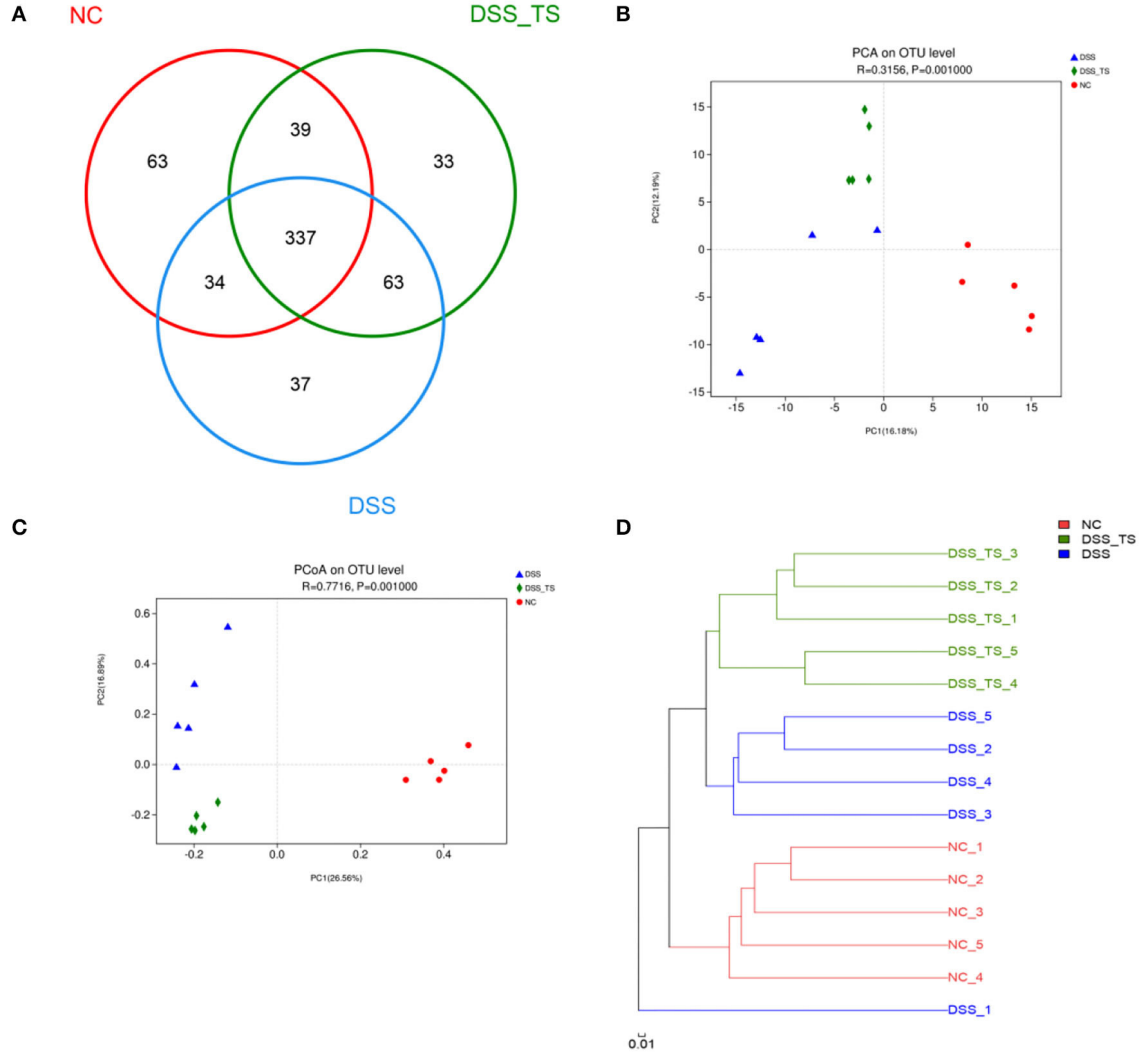
TS modulates the gut microbiota composition induced by DSS treatment. **(A)** OTU number in each group; **(B)** PCA of gut microbiota communities based on OTU level; **(C)** PCoA of gut microbiota communities based on OTU level; **(D)** System clustering tree of gut microbiota based on unweighted Unifrac metrics indicating the beta diversity of gut microbiota in each group.

To determine the impact of TS on the gut microbiota in DSS-induced colitis, we detected the gut microbiota by 16s rRNA sequencing. There was no significant difference in the OTU number between the NC, DSS, and DSS+TS groups (Figure 6A). The β-diversity analysis showed a distinct demarcation in microbial composition between the three groups, including the principal components analysis (PCA), principal coordinate analysis (PCoA) and the system clustering tree (Figures 6B–D). The PCA (ANOSIM: *R* = 0.3209, *p* = 0.0010) and PCoA (ANOSIM: *R* = 0.7716, *p* = 0.0010) of Bray-Curtis based on OTUs presented that DSS changed the gut microbiota significantly, while TS could regulate the gut microbiota (Figures 6B,C). The system clustering tree demonstrated the same result that DSS+TS group was clustered separately from DSS group (Figure 6D). However, TS only changed some microbiome and had little effect on the composition of total gut microbiota, so DSS-TS microbiome was more similar to the DSS group than the NC group.

**Figure 7:**
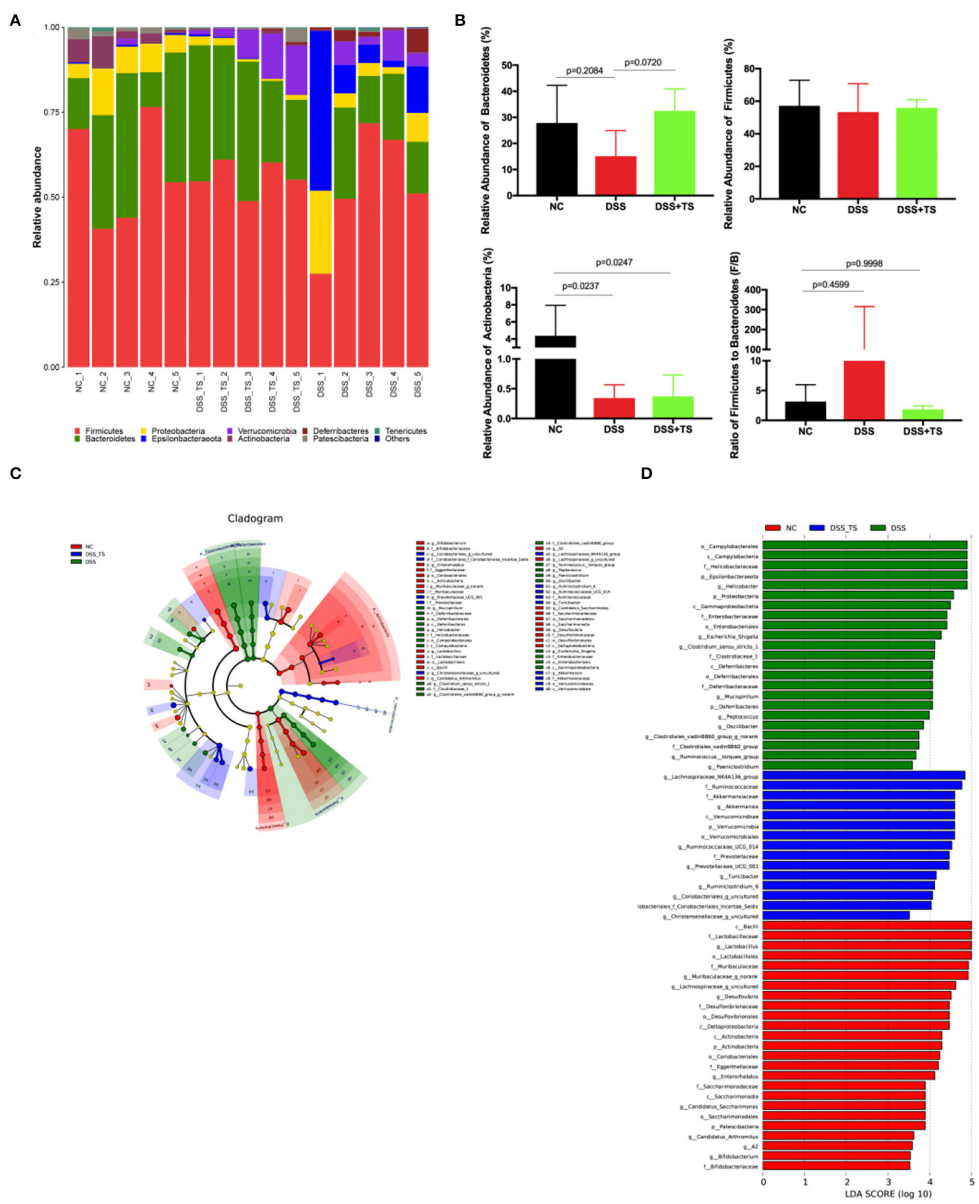
Gut microbiota composition was altered by DSS and TS intervention according to LEfSe analysis. **(A)** Column diagram of the microbial composition at phylum level; **(B)** the relative abundances of *Bacteroidetes, Firmicutes* and *Actinobacteria*, the ratio of *Firmicutes* to *Bacteroidetes*; **(C)** taxonomic cladogram obtained from LEfSe sequence analysis; **(D)** Taxa with a different abundance in each group. Data are expressed as mean ± SEM, statistical significance was determined with one-way ANOVA followed by Tukey test.

Microbial community histogram disclosed the compositions of the intestinal microbiota at a phylum level (Figures 7A,B). The detected phyla included *Firmicutes, Bacteroidetes, Proteobacteria, Actinobacteria, Verrucomicrobia*, and *Deferribacteres*. In the DSS group, the abundance of *Bacteroidetes* was decreased compared with the NC group. The modification in *Bacteroidetes* also resulted in an increase in the F/B ratio in the DSS group compared with the NC group. While the abundance of *Bacteroidetes* was increased and the F/B ratio was reduced in DSS+TS group. We also noted Actinobacteria, which was significantly more abundant in the NC group, than in the DSS and DSS+TS groups. The linear discriminant analysis effect size (LEfSe) method was employed to identify the characteristic bacterial taxa among these groups (Figures 7C,D). In the cladogram, the key bacterial taxa for each group was marked with relevant colorful nodes at the levels of phylum, class, order, family and genus (Figure 7C). The prominent bacterial taxa for each group was also displayed according to the LDA scores in the LEfSe Bar (Figure 7D). The results demonstrated that the predominant bacteria in the DSS group contained several pathogens like *Campylobacteria, Proteobacteria, Helicobacter*, and *Enterobacteriaceae*. Additionally, some probiotics like *Akkermansia, Bacteroides*, and *Lactobacillus* were increased in DSS+TS groups compared with DSS group, suggesting the positive effect of TS on the intestinal microbiota.

### TS Treatment Changed the Metabolic Genes

**Figure 8:**
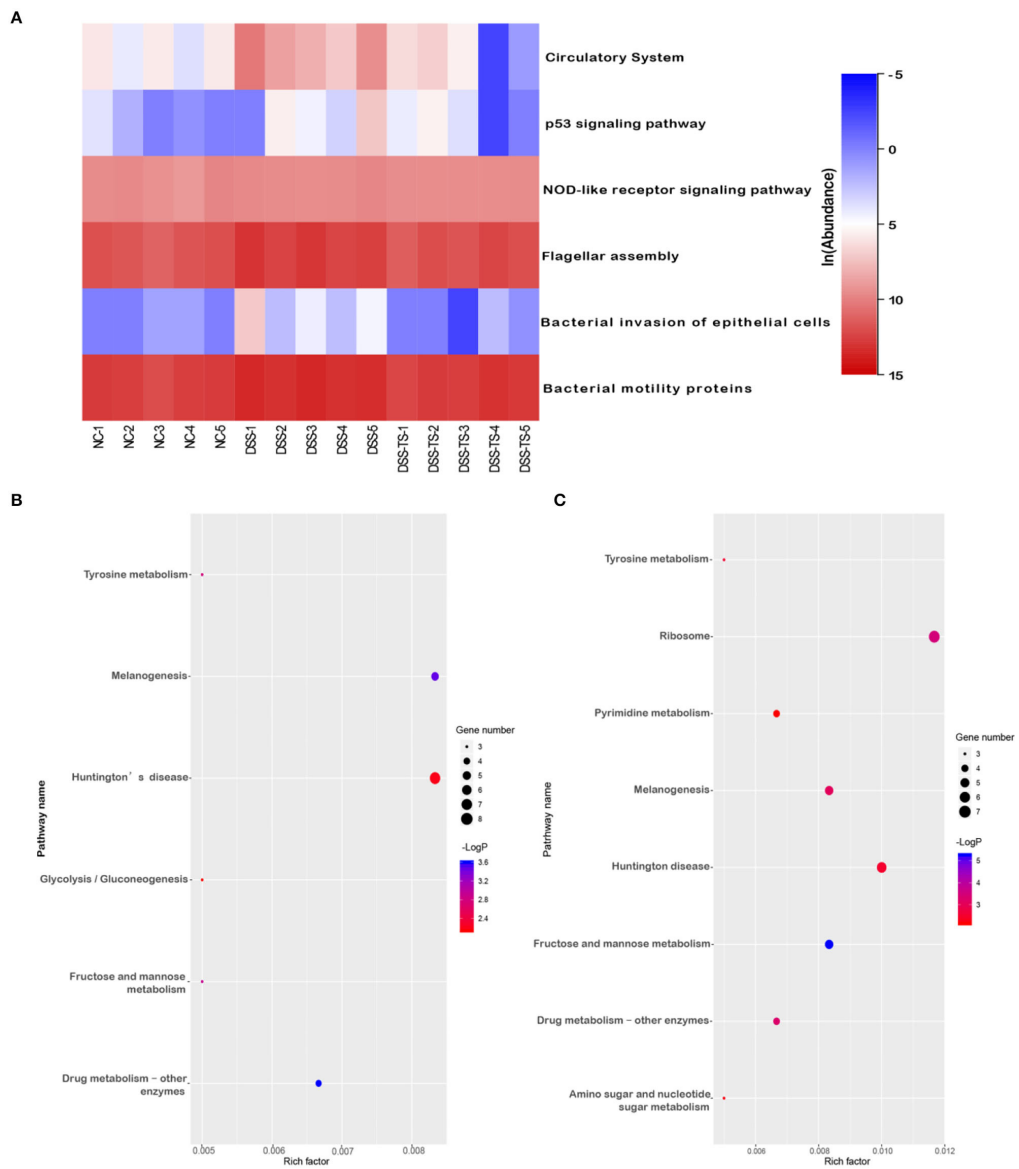
The effect of TS on the microbial community function predicted by PICRUSt. **(A)** Heatmap shows the relative abundance of pathways in different groups; Bubble chart shows the gene abundances in pathways **(B)** DSS group relative to NC group, **(C)** DSS+TS group relative to DSS group.

Besides the microbiota community, the discrepancy of functional profiles between different groups predicted by PICRUSt software is exhibited in Figure 8. The investigation of communities by PICRUSt was used in our work and the predicted metagenome information was then collapsed into the KEGG pathway (level 3) based on the 16S rRNA sequences. SangerBox package was used for functional profiling. In total, the treatment of DSS mainly caused changes in 6 pathways (Figure 8A), including the circulatory system, p53 signaling pathway, NOD-like receptor signaling pathway, flagellar assembly, bacterial invasion of epithelial cells, and bacterial motility proteins. Compared with the NC group, DSS treatment promoted these pathways, while these pathways were down-regulated after TS treatment. Meanwhile, the treatment of DSS caused changes in some genes and the gene abundances in pathways of drug metabolism, tyrosine metabolism, glycolysis/gluconeogenesis, fructose and mannose metabolism, melanogenesis, Huntington's disease (Figure 8B). TS treatment led to changes in more complex functional pathways, mainly in fructose and mannose metabolism, pyrimidine metabolism, amino sugar and nucleotide sugar metabolism, ribosome and human diseases such as melanogenesis, Huntington's disease (Figure 8C). Therefore, TS intervention contributed to the functional difference of the gut microbiota.

### The Effect of Gut Microbiota Induced by TS in DSS-Induced Colitis in Mice

**Figure 9:**
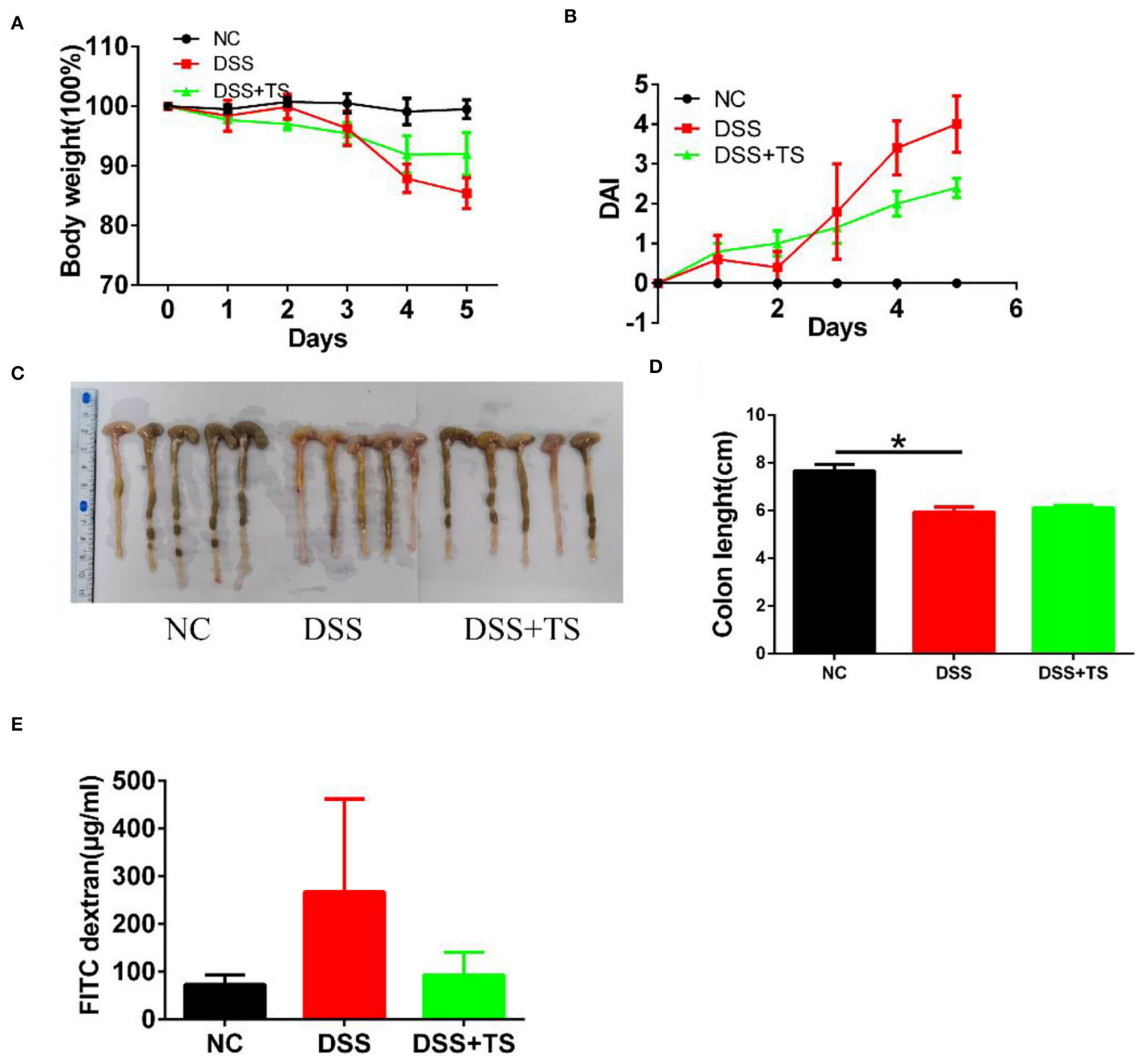
The effect of microbiota induced by TS in DSS-induced colitis in mice. **(A)** Body weight; **(B)** DAI score; **(C)** Images of colon; **(D)** colon length; **(E)** detection of FITC-dextran in serum. Data are expressed as the mean ± SEM (*n* = 5), statistical significance was determined with one-way ANOVA followed by Tukey test, and *p*-values are as follows: ^*^p < 0.05.

To confirm the important role of microbiota induced by TS, we conducted the fecal transfer experiment. We took the feces of the NC group, DSS group and DSS+TS group, and administered them to antibiotic-treated sterile mice by orally gavage for 5 consecutive days. The mice treated with gut microbiota of DSS+TS group showed a reduced DSS damage, including body weight loss and a lower DAI scores in the group that gut microbiota administered by DSS+TS group compared with the DSS group (Figures 9A,B). The general appearance and length of the colon were not significantly different between the DSS group and the DSS+TS group in Figures 9C,D. In addition, the diffusion of FITC-dextran from the intestine to the serum after gut microbiota induced by TS treatment was significantly decreased compared with the DSS group (Figure 9E), suggesting that gut microbiota induced by TS improved the ability of maintaining the function of the colonic mucus barrier during DSS administration.

### The Association of Gut Microbiota Induced by TS With Gut Inflammation

**Figure 10:**
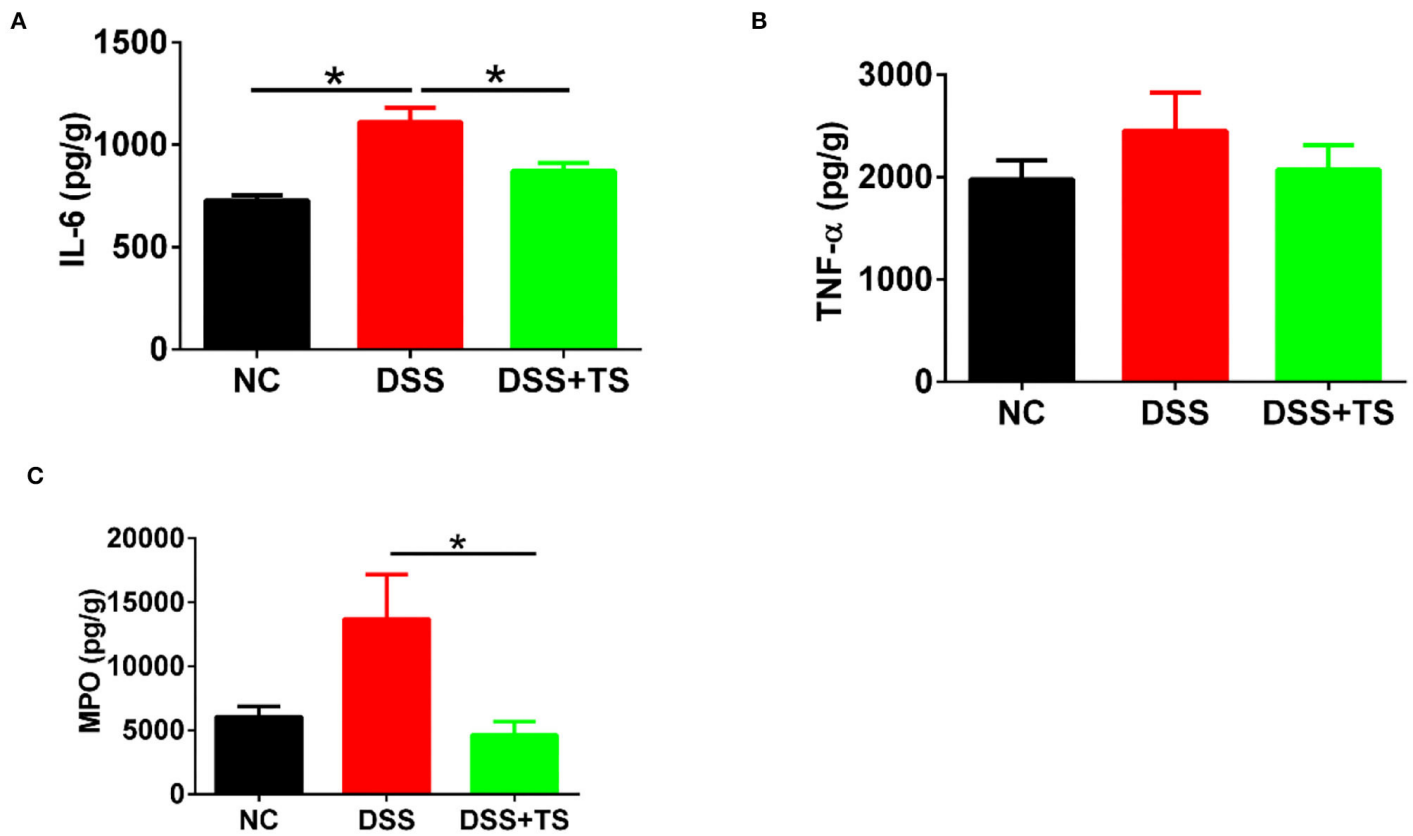
The association of microbiota induced by TS with gut inflammation. **(A,B)** the protein levels of IL-6 and TNF-α by ELISA; **(C)** the protein levels of MPO by ELISA. Data are expressed as mean ± SEM (*n* = 5), statistical significance was determined with one-way ANOVA followed by Tukey test, and *p*-values are as follows: ^*^p < 0.05.

We demonstrated that TS ameliorated murine colitis through the modulation of immune response, mucus barrier and gut microbiota, thus improving gut dysbiosis, However, there was no relation analysis between the immune response and gut microbiota. We took the feces of the NC group, DSS group and DSS+TS group, and administered them to antibiotic-treated sterile mice. The mice treated with gut microbiota of TS treatment showed a reduced level of IL-6 (Figure 10A), TNF-α (Figure 10B), and MPO (Figure 10C) compared with the DSS group, however, TNF-α showed no statistical difference.

## Discussion

DSS treatment was reported to shorten the length of the colon and increased DAI scores including weight loss, diarrhea and hematochezia in mice (14), while TS intervention increased body weight, ameliorated bloody stools, increased the colon length and decreased the DAI score, indicating that TS exerted an anti-inflammatory effect on DSS-induced colitis and might have potential to prevent and cure IBD patients.

Induced activation of p65 in response to a variety of stimuli is typically transient but sufficient to upregulate transactivation of target genes of diverse activities such as the cellular proliferation, inflammatory cytokines (15, 16). Consequently, TS inactivated NF-κB pathway signaling, leading to the decline of inflammatory cytokines and finally promoting the intestinal physiology and pathophysiology. The production of TNF-α, IL-1β, and IL-6 plays leading roles in the initiation and progression of colitis, and some of them have been regarded as a logical target for IBD therapy (8, 17). Thus, we hypothesized that TS attenuated the inflammatory response in the gut by inhibiting NF-κB activation.

IL-6 is released upon stimulation of inflammatory cells to activate signaling pathways such as STAT3 (18). STAT3 is activated in both the epithelial and hematopoietic compartments in IBD (19, 20). Consistently, our results showed that STAT3 was more activated in the DSS group compared with DSS+TS group. The role of STAT3 in the epithelial compartment is complex, it is important for inducing return to homeostasis following acute infection, while at the same time it involves the induction of cytokines and chemokines expression. Perhaps, TS suppressed inflammatory cytokines partly via inactivating STAT3 in DSS-induced colitis mice.

Colitis is an immune-related disease caused by neutrophil and macrophage infiltration in the colonic lamina propria (21, 22), but the effect of TS on immune cells is unclear. Our study found that dietary TS ameliorated neutrophil accumulation in the colon of DSS-induced colitis mice which was accompanied with decreased TNF-α, IL-6, and IL-1β expression. The microbiota of IBD patients can change the intestinal CD4+T cell homeostasis of the sterile recipient mice (23). Our results also demonstrated that colonization with CD4+T cells was dramatically increased in DSS-induced colitis, and TS inhibited the recruitment of CD4+T cells. In conclusion, TS mainly regulates neutrophil infiltration to protect mice against DSS-induced colitis.

Besides immune system disorders, one typical symptom of IBD is weakening of the mucus barrier, leading to further gut bacterial disruption and inappropriate immune activation (24). The colonic epithelium contains several differentiated cell types like enterocytes and goblet cells, each of which responsible for absorption, mucus production and antigen sampling (25, 26). Defects in mucus production and tight junction have been described in IBD (7, 27, 28). DSS-induced colitis was also found to be associated with the decreased expression of Muc2, Claudin-1, and ZO-1. TS significantly ameliorated these markers, which could preserve the mucus barrier away from DSS invasion via decreasing pro-inflammatory cytokines levels. Relevant studies reported the possible mechanisms and even considered that the intestinal integrity might be a crucial target for the treatment of IBD (29).

Gut microbiota is increasingly thought of as a key factor in the cause and therapy target of IBD. The immune system can be triggered by gut microbiota, showing a cause-and-effect relationship with the disease (30). IBD has been shown to be associated with change in the composition and metabolism of the gut microbiota due to a shift between commensal and pathogenic microorganisms (31, 32). Although there is no conclusive direct causal relationship between IBD and dysbiosis, some specific bacteria have abilities to induce pro-inflammatory or protective effects and the chronic inflammation has access to shape the gut microbiota (33–35). The underlying mechanisms still need further study. In this study, we found that the structure and composition of the gut microbiota underwent changes in mice with DSS-induced colitis. At the phylum level, the F/B ratio was increased, which is consistent with the finding of other studies (7, 36, 37). TS could reverse the F/B ratio to correct the disorder of the intestinal microbiota induced by DSS. As inflammation is an oxidative state, it might be expected to promote the outgrowth of aerotolerant taxa such as Actinobacteria (38). A cohort study reported a significant increase in Actinobacteria in UC patients (39). Whereas, it has been reported previously in IBD that there is a decrease in Actinobacteria abundance compared to healthy subjects (40). Therefore, previous reports on the abundance of actinomycetes were not consistent in IBD patients. Since in our analysis the relative abundance of Actinobacteria was also significantly increased in DSS-induced colitis, this aspect should be further investigated.

Additionally, the DSS group showed higher abundance of pathogenic microbiota (e.g., *Campylobacteria, Proteobacteria, Helicobacter*, and *Enterobacteriaceae*) and lower beneficial microbiota (e.g., *Lactobacillus* and *Bifidobacterium*) compared with the NC group in our study. *Campylobacteria* has emerged as a putative player in the pathogenesis of IBD due to its extraordinary and diverse pathogenic capabilities (41). *Proteobacteria* is considered as a typical pathogen associated with IBD pathogenesis (37). Lupp et al. reported that colonic inflammation supported the growth of some specific aerobic bacteria in the gut, especially the *Enterobacteriaceae* family (42). In our research, the *Enterobacteriaceae* family was abundant and even *Escherichia-Shigella* became the dominant bacteria in the DSS group. *Shigella* is considered the crucial factor leading to the increased intestinal permeability and even worsening the disease (43, 44) *Akkermansia muciniphila* is known as a member of *Akkermansia* with the ability to maintain the intestinal integrity and exhibit anti-inflammatory responses (45, 46).

Meanwhile, significantly different functional profiles between different groups were predicted by PICRUSt to highlight the importance of the gut microbiota in these metabolic pathways. Our results showed relatively high abundances in flagellar assembly, bacterial invasion of epithelial cells and bacterial motility proteins in the DSS group, which could be significantly reversed by TS treatment. These pathways play important roles in bacteria adhesion and motility. Adhesion to mucus and invasion of epithelial cells are essential for infection by pathogenic bacteria. Studies have shown that infection with pathogenic bacteria such as *Salmonella* and *C. difficile* promotes the onset of IBD (47, 48). In TS treatment colitis, a decrease in the identification of predictive microbial signals could also allow for refinement of TS that may deliver specific anti-inflammatory role that may be of benefit in ameliorating inflammation. Therefore, the inhibitory effect of TS on bacterial adhesion and invasion to the host may have a therapeutic value in IBD. The predominantly glycolytic form of metabolism shared by Polymorphonuclear leukocytes (PMNs) is thought to ensure their survival and function in the hypoxic, often anoxic environment of deep inflammatory foci. In DSS-induced colitis, the increase of glycolytic may reflect the recruitment of PMNs. It was found in the present study that the intervention of TS significantly improved this function. In addition, compared with the DSS group, TS treatment led to changes in more complex functional pathways, such as pyrimidine metabolism, amino sugar and nucleotide sugar metabolism and ribosome. These results demonstrated the ability of TS from tea to adjust metabolic pathways of the gut microbiota in IBD, though further experiments are necessary to confirm identification of these pathways and related genes.

In conclusion, our study demonstrated that DSS treatment disturbed the gut microbiota by triggering an immune response, and TS treatment significantly attenuated the severity of inflammation, modulated the metabolism pathways and restored the unbalanced microbiota composition to a normal condition in mice with DSS-induced colitis. In addition, we delineated the features of the intestinal microbiota that are associated with IBD. Although our study demonstrated an association between TS treatment and change of the microbiota, causative studies are needed to completely understand the role of the gut microbiota during TS treatment. Furthermore, our study provided the better understand the role of the gut microbiota of TS treatment in controlling the induction of immune system in mice with DSS-induced colitis. Our data also provide a foundation for future study at the transcriptional level to better establish the metabolomics profiles associated with IBD. This study adds to our understanding of interactions between the immune system, the microbiota and the host, and provides the basis for future clinical study of microbiota manipulation and the development of new therapeutic strategies for IBD.

## Data Availability Statement

The original contributions presented in the study are included in the article/supplementary materials, further inquiries can be directed to the corresponding author/s.

## Ethics Statement

The animal study was reviewed and approved by Laboratory Animal Ethics Committee of Xuzhou Medical University (201908A005).

## Author Contributions

HY is responsible for providing the overall idea. RC drafted the paper. ZK and SQ are in charge of experimental design. YC is responsible for data collation. CC is in charge of animal experiments. BG made important changes to the paper. All authors contributed to the article and approved the submitted version.

## Conflict of Interest

The authors declare that the research was conducted in the absence of any commercial or financial relationships that could be construed as a potential conflict of interest.
